# The gut–brain connection: microbes’ influence on mental health and psychological disorders

**DOI:** 10.3389/frmbi.2025.1701608

**Published:** 2026-01-07

**Authors:** Pegah Ataei, Hamidreza Kalantari, Tamara S. Bodnar, Raymond J. Turner

**Affiliations:** 1 Department of Clinical Psychology, Islamic Azad University, East, Azarbaijan, Iran; 2 Department of Microbiology, Islamic Azad University, Tehran, Iran; 3 Microbial Biochemistry Laboratory, Department of Biological Sciences, University of Calgary, Calgary, AB, Canada

**Keywords:** gut–brain axis, mental health, microbiome, microbiome nexus, neurotransmitters and microbiota, psychological disorders

## Abstract

The human gut microbiome has emerged as a pivotal modulator of brain function and mental health, acting through intricate bidirectional communication along the gut–brain axis. Mounting evidence suggests that microbial communities influence neurodevelopment, neurotransmission, and behavior via pathways involving the vagus nerve, immune signaling, and microbiota-derived metabolites such as short-chain fatty acids and neurotransmitter precursors. This review critically examines the mechanistic underpinnings of microbiota–brain communication and evaluates current findings linking dysbiosis to psychiatric conditions, including depression, anxiety, schizophrenia, autism spectrum disorder, and bipolar disorder. In addition, it assesses the therapeutic potential of microbiome-targeted interventions—such as probiotics, fecal microbiota transplantation (FMT), and precision dietary modulation—in ameliorating neuropsychiatric symptoms. While the field holds considerable promise, limitations, including correlational study designs, small sample sizes, and a lack of standardized methodologies, underscore the need for rigorous, large-scale clinical trials. A deeper understanding of host–microbe interactions may catalyze a paradigm shift in psychiatric treatment, paving the way for novel, personalized microbiome-based therapeutics.

## 1 Introduction

**Figure 1:**
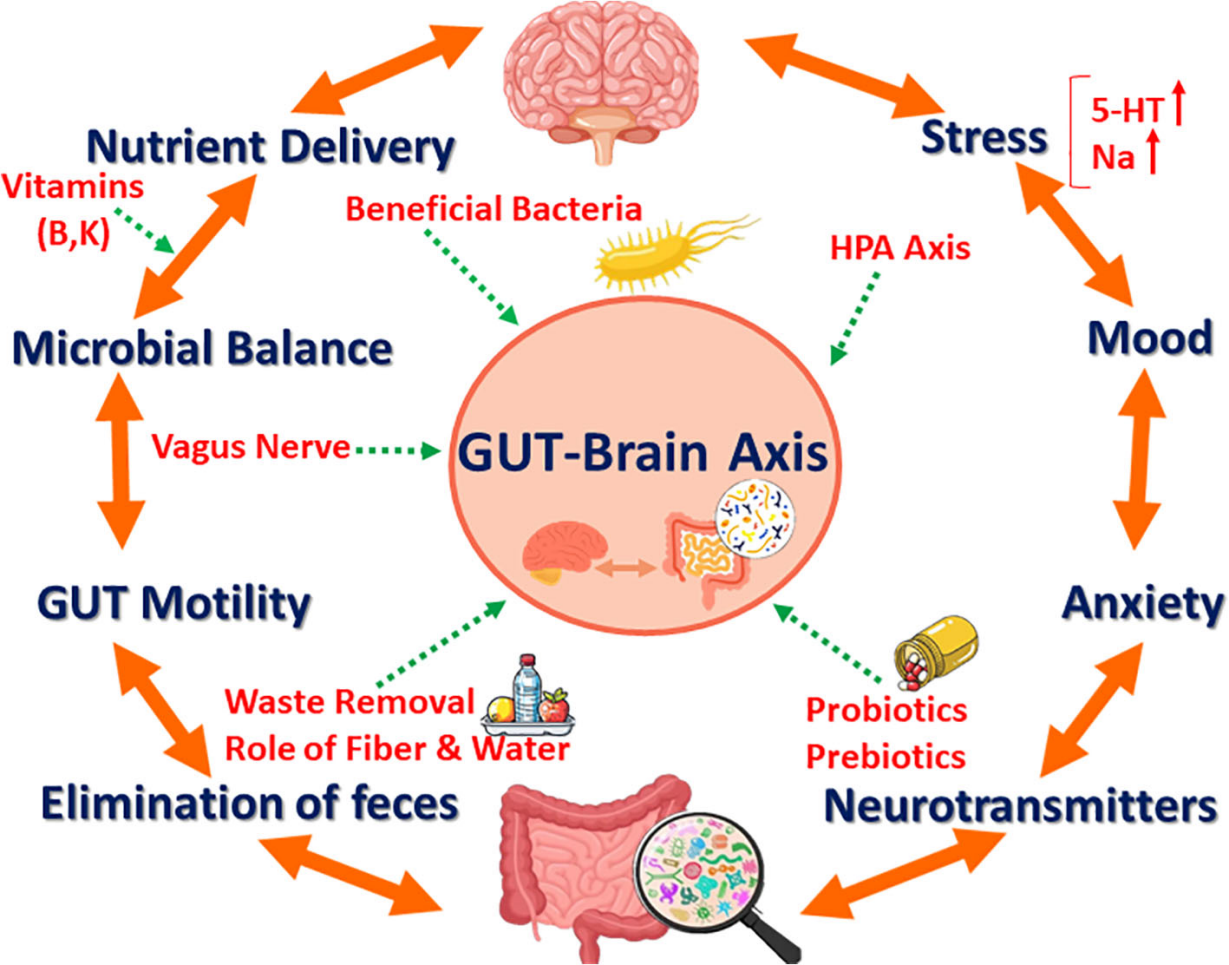
The gut–brain axis. The gut–brain axis involves communication between the gastrointestinal tract and the brain, influencing emotional and physiological processes through microbial balance and nutrient delivery.

The human body hosts trillions of microorganisms, collectively known as the microbiome, which are essential for maintaining physiological balance (Patel, 2021). While their roles in digestion and immunity are well established, emerging research indicates that microbes also significantly influence brain function and behavior, with increasing implications for mental health (Singh et al., 2022). A growing body of research suggests that the microbiome plays a significant role in mental health, potentially affecting mood, cognitive function, and even the risk of developing neurological disorders (Smith and Wissel, 2019). The gut–brain network (**Figure 1**) a complex bidirectional communication network linking the gastrointestinal system and the central nervous system, has recently emerged as a key area of interest in the fields of neuroscience and psychiatry. The gut–brain axis involves multiple pathways, including neural, hormonal, and immune signaling, which collectively influence brain function, emotional regulation, and overall mental health (Mayer et al., 2022). As researchers delve deeper into this dynamic relationship, there is growing recognition of how the gut microbiota may contribute to neurological and psychiatric conditions, opening doors to novel therapeutic approaches (Bhatia et al., 2023). Psychological disorders, including depression, anxiety, schizophrenia, and autism spectrum disorder (ASD), have been increasingly linked to imbalances in the gut microbiota, a phenomenon known as dysbiosis (Meng et al., 2020). Research indicates that disruptions in gut microbial community diversity, bacterial abundance and composition can trigger a cascade of physiological changes, including heightened neuroinflammation, increased oxidative stress, and imbalances in neurotransmitter signaling—factors that are closely associated with the development and progression of psychiatric illnesses (Belizário et al., 2018). Mechanistically, compositional shifts often reduce populations of short-chain fatty acids (SCFA)-producing and barrier-supporting taxa while enriching endotoxin-producing groups; this imbalance can compromise intestinal barrier integrity and increase translocation of microbial products (e.g., lipopolysaccharide) and atypical metabolites into the circulation. These peripheral signals activate systemic immune pathways, promote microglial priming and neuroinflammation, increase oxidative stress, and alter host metabolic routing of precursors (e.g., tryptophan → kynurenine), all of which may perturb neurotransmitter systems and neuroplasticity. Importantly, the directionality of these associations remains unresolved: causal inference in humans is limited by the predominance of cross-sectional studies, although experimental approaches such as fecal microbiota transfer in animal models and a small number of longitudinal and interventional studies provide partial evidence that microbiota alterations can drive behavioral and neurochemical changes. Thus, while mechanistic pathways linking dysbiosis to brain physiology are increasingly well characterized, further longitudinal and controlled human studies are required to establish causality (Chelakkot et al., 2018; An et al., 2022). Conversely, growing evidence suggests that targeted interventions aimed at restoring microbial “balance”, such as probiotics, prebiotics, and dietary modifications, may help with dysbiosis and offer promising therapeutic strategies for enhancing mental well-being and mitigating symptoms of neuropsychiatric disorders (Meher et al., 2024; Zhou et al., 2024).

An emerging area of interest in microbiome research is its role in early brain development and lifelong mental health. Studies suggest that the composition of the gut microbiota is shaped from birth and continues to evolve in response to diet, the environment, and lifestyle (Rodríguez et al., 2015). Disruptions during critical developmental periods—such as birth by cesarean section, lack of breastfeeding, antibiotic use, or early-life stress—have been linked to an increased risk of neuropsychiatric disorders later in life (Cho, 2021; Green et al., 2025). For example, research has shown that germ-free mice exhibit exaggerated stress responses and cognitive deficits, reinforcing the idea that the microbiome is essential for normal brain development and function (Luczynski et al., 2016). Understanding how microbial communities establish and influence neurodevelopment could open new avenues for early interventions aimed at preventing or treating disorders that often coincide with mental health challenges.

This review seeks to provide an overview of the intricate and dynamic relationships between the microbiome and psychological disorders, shedding light on the wide range of ways in which microbes may influence brain function and mental health. The underlying mechanisms through which the gut microbiota impacts development, neurological processes, and progression of various psychiatric conditions will be examined, highlighting the potential for promising novel, microbiome-based therapeutic strategies in this field.

## 2 Interactions between the gut microbiome and the nervous system

**Figure 2:**
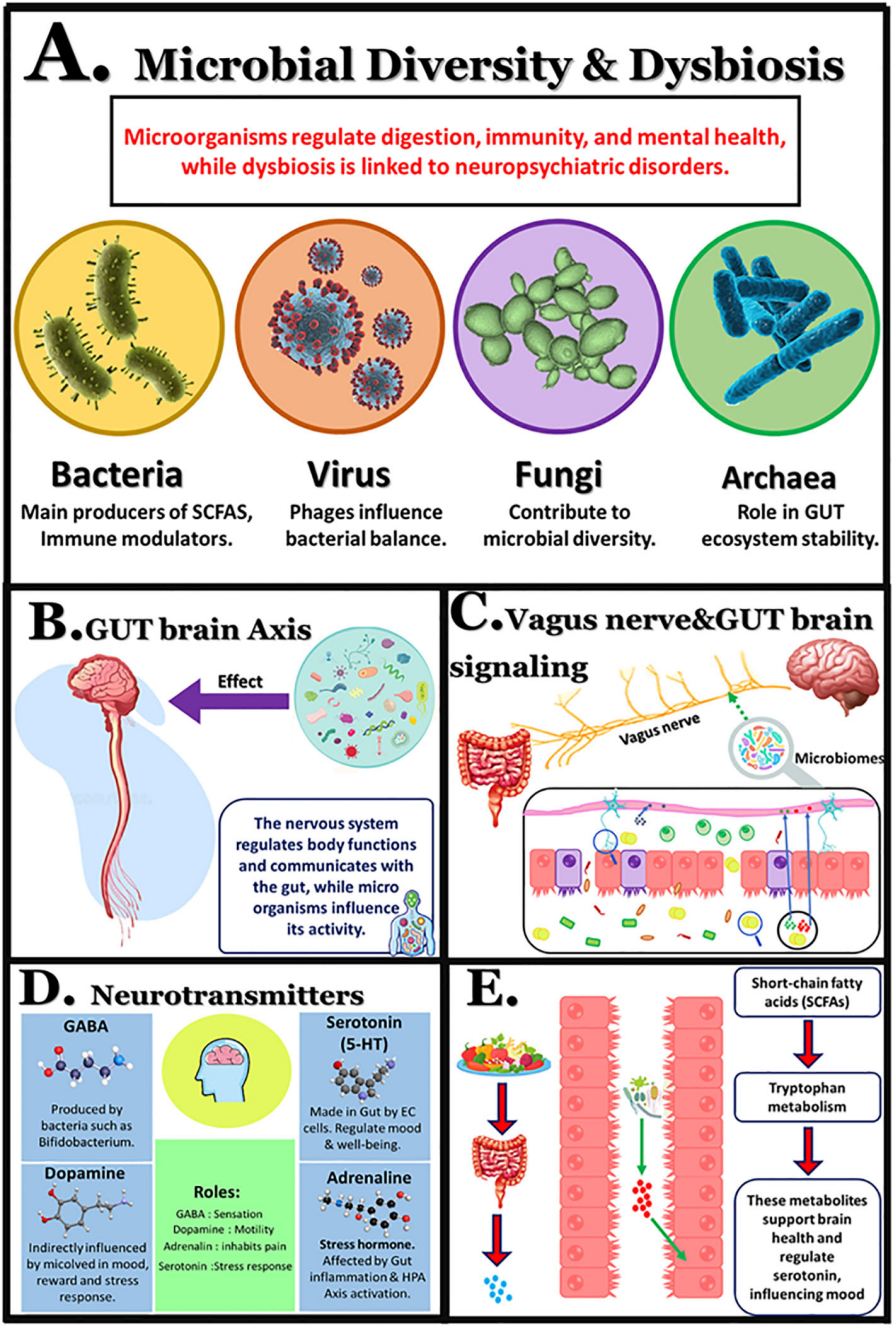
The microbiome and nervous system. **(A)** Major microbial groups influencing health; **(B)** Bidirectional communication between gut microbes and the brain; **(C)** Gut–brain signaling via the vagus nerve and intestinal barrier; **(D)** Microbial modulation of neurotransmitters; **(E)** Role of microbial metabolites, including SCFAs and tryptophan derivatives, in mood regulation.

The microbiome and nervous system (**Figure 2**) communicate through the gut–brain axis, influencing mood, cognition, and behavior. Gut microbes have been shown to directly or indirectly impact neurotransmitters, inflammation, the immune system and neural signaling, ultimately having the potential to impact mental health and neurological function.

### 2.1 Microbiome

The gastrointestinal tract harbors trillions of microbial cells that produce key metabolites such as SCFAs and vitamins, which contribute to digestion, immune regulation, and gut barrier integrity (Lipski, 2020; Lee et al., 2022). These microbes’ impact metabolic functions and produce essential biomolecules that include SCFAs, vitamins such as menaquinone (vitamin K2), a bacterially produced form of vitamin K2 (Ellis et al., 2021), and neurotransmitters that contribute to the maintenance of the integrity of the gut barrier. Some clinical studies have suggested that supplementation with vitamin K2 may play a role in the treatment of depression, potentially through its involvement in anti-inflammatory and neuroprotective pathways (Liu et al., 2022; Tarkesh et al., 2022; Wang S.-T. et al., 2024). Notably, certain gut microbiota species can produce vitamin K2 within the colon, suggesting that microbiome composition may indirectly influence neurological health by contributing to the local synthesis of neuroactive vitamins. Therefore, a healthy microbiome is characterized by diversity and balance, and disruptions—commonly referred to as dysbiosis—have been linked to various health conditions (Fassarella et al., 2021).

### 2.2 Nervous system: structure and function

The nervous system, which is composed of central and peripheral branches, plays a central role in regulating behavior, cognition, and vital physiological processes (Sabina et al., 2024). Increasing evidence highlights a strong, bidirectional relationship between the nervous system and the gastrointestinal tract, known as the gut–brain axis (Arneth, 2018). This connection is mediated by complex signaling networks involving the vagus nerve, neurotransmitters, immune signals, and microbial metabolites produced in the gut. The enteric nervous system, often referred to as the “second brain,” operates semi independently but remains in constant communication with the brain, allowing gut microbes to influence neural processes such as mood regulation, stress response, and neurodevelopment (Garzone et al., 2025). Understanding this dynamic interaction offers critical insights into how the gut microbiota may contribute to both neurological and psychological conditions. Bidirectional communication between the gut and the brain is facilitated by signaling pathways involving electrical impulses; neuroactive compounds such as serotonin, dopamine, and gamma-aminobutyric acid (GABA); and immune modulators such as cytokines and SCFAs, which are bacterial products. Gut–brain communication occurs through multiple interconnected pathways, including the vagus nerve, the immune system, microbial metabolites such as SCFAs, and endocrine signaling. These mechanisms enable the gut microbiota to influence neural activity, mood regulation, and stress responses. Immune signaling plays a key role in this axis, as the gut microbiota regulates systemic inflammation and immune cell activity, which can in turn affect brain function. This complex neurochemical and immunological crosstalk—often referred to as the gut–brain–immune axis—is a fundamental component of host physiology, highlighting the biological relevance of microbial communication beyond the gut.

### 2.3 Communication via the vagus nerve

The vagus nerve, the longest cranial nerve in the body, serves as a key communication pathway between the gut and the brain (Wang Y. et al., 2024). The vagus nerve enables bidirectional signaling between the gut microbiota and the CNS, allowing microbial activity to influence brain function and behavior (Liu and Forsythe, 2021). While vagus nerve stimulation has been shown to alleviate symptoms of depression and anxiety, it is not direct evidence of microbiome involvement (Karemaker, 2022). However, preclinical studies suggest that the vagus nerve may serve as a key communication route by which gut microbes influence brain function, linking microbial signals to neural and emotional regulation (Kong et al., 2018). In addition, certain probiotic strains—often termed “psychobiotics”—can modulate the gut microbiota composition and metabolic activity, enhancing the production of neuroactive compounds such as GABA, serotonin precursors, and SCFAs. These metabolites interact with enteroendocrine and epithelial receptors in the gut, stimulating afferent signaling via the vagus nerve and enteric nervous system (ENS) (Smith et al., 2021; Deng et al., 2024). Animal studies have demonstrated that the effects of probiotics on mood and behavior are abolished after subdiaphragmatic vagotomy, confirming this neural route (Needham et al., 2022). Metabolites such as SCFAs also act through free fatty acid receptors (e.g., FFAR2/3), modulating microglial activation, neuroinflammation, and neurotransmitter systems, further influencing emotional regulation (Mishra et al., 2020). Finally, these microbial signals may help restore stress axis balance by reducing hypothalamic pituitary adrenal (HPA) axis hyperactivity and proinflammatory cytokines—effects supported by rodent and early human clinical trials (Rusch et al., 2023).

### 2.4 Gut microbiota and neurotransmitters

Gut microbes play pivotal roles in the production and regulation of neurotransmitters—chemical messengers that influence mood, cognition, and behavior (Mittal et al., 2017). Several key neurotransmitters are either synthesized directly by gut bacteria or modulated through microbial activity:

#### 2.4.1 Serotonin

Approximately 90% of the body’s serotonin—a neurotransmitter essential for regulating mood, cognition, and emotional behavior—is synthesized in the gastrointestinal tract. Resident gut bacterial strains, notably *Lactobacillus* and *Bifidobacterium*, play important roles in modulating serotonin levels by affecting the metabolism of tryptophan, the primary precursor of serotonin (Corano Scheri et al., 2017; Jeong et al., 2021). These bacteria can shift tryptophan away from the kynurenine pathway—often activated under stress and inflammation—towards the serotonergic pathway, increasing peripheral serotonin synthesis (Correia and Vale, 2022). Moreover, tryptophan metabolism by gut microbes involves the enzymatic activity of tryptophanase, which converts tryptophan into indole and related metabolites. Importantly, indole compounds have been shown to influence host neuroendocrine pathways, immune modulation, and intestinal barrier integrity (Ye et al., 2022). Furthermore, indole and its derivatives are implicated in the regulation of biofilm formation in gram-negative bacteria (Minvielle et al., 2013), thereby affecting microbial colonization and the ecological balance of the gut (Ye et al., 2022). These biochemical pathways also interact with the synthesis of other critical neuroactive compounds, such as dopamine and tyrosine-derived catecholamines (described below). Disruptions in the production and regulation of these compounds can lead to neurochemical imbalances associated with various neuropsychiatric conditions (Stasi et al., 2019; Simpson et al., 2021). For example, indole-3-propionic acid (IPA), a tryptophan-derived microbial metabolite, has been shown to exert neuroprotective effects by reducing oxidative stress and modulating inflammatory responses in the brain (Konopelski and Mogilnicka, 2022; Kim et al., 2023). Reduced levels of IPA have been observed in patients with depression and stress, suggesting that diminished microbial production of protective indole derivatives may contribute to the pathophysiology of these disorders (Konopelski and Mogilnicka, 2022; Mu et al., 2025). Thus, the interplay between microbial tryptophan metabolism and host neurotransmitter systems appears to be at a connection between the gut microbiota and mental health outcomes.

#### 2.4.2 Dopamine

Dopamine is associated with motivation, reward, and emotional regulation and is influenced by CNS activity as well as the metabolic capacity of the gut microbiota. Although dopamine itself cannot cross the blood–brain barrier, the gut can produce significant amounts of its biochemical precursors, which may influence systemic physiology and indirectly affect brain function. Several gut-residing bacteria, including *Escherichia coli, Bacillus subtilis, Proteus vulgaris, Serratia marcescens*, and *Staphylococcus aureus*, have been shown to produce dopamine or its precursors, particularly tyrosine and L-DOPA (levodopa) (Oleskin et al., 2021; Eicher and Mohajeri, 2022). These precursors are central intermediates in dopamine biosynthesis, with tyrosine hydroxylated into L-DOPA, which is then decarboxylated to form dopamine. Species such as *Enterococcus faecalis* and *Enterococcus faecium* express tyrosine decarboxylase enzymes capable of directly converting dietary L-DOPA into dopamine within the gut lumen (Villageliú and Lyte, 2018). This activity has clinical relevance, especially in the context of neurological disorders such as Parkinson’s disease, where gut microbial metabolism can influence the bioavailability of therapeutic L-DOPA—the precursor to dopamine that must cross the blood–brain barrier to affect central nervous system function (Niehues and Hensel, 2009; Van Kessel and El Aidy, 2019). Moreover, microbially derived dopamine can interact with the enteric nervous system, potentially modulating gastrointestinal motility and immune signaling by acting on dopamine receptors expressed on immune cells, such as T cells and macrophages, thereby affecting cytokine production and inflammatory responses within the gut (Levite, 2016; Scott et al., 2024). Overall, alterations in microbial populations that affect dopamine-related pathways may contribute to the severity and progression of neuropsychiatric conditions such as Parkinson’s disease and schizophrenia by influencing neurotransmitter availability and neuroinflammatory processes, highlighting the functional relevance of the gut–brain axis in maintaining mental health. Further research is needed to determine the extent to which these peripheral pathways influence central dopaminergic signaling.

#### 2.4.3 Gamma-aminobutyric acid

GABA is the primary inhibitory neurotransmitter in the CNS; it is crucial for regulating neuronal excitability and is involved in the modulation of anxiety, stress responses, and mood regulation. Emerging evidence indicates that certain gut microbes possess the enzymatic machinery required to synthesize GABA from glutamate via glutamate decarboxylase (GAD). Among the most well-documented GABA-producing strains are *Lactobacillus rhamnosus*, *Lactobacillus brevis*, and *Bifidobacterium dentium*. These microbes are capable of producing GABA within the gut lumen, where it may exert effects locally on the ENS or indirectly via the vagus nerve and subsequently impact the CNS (Tette et al., 2022). Preclinical studies, particularly in murine models, have demonstrated that *L. rhamnosus* (JB-1) administration alters GABA receptor expression in specific brain regions, such as the amygdala and hippocampus, and that this effect is correlated with reduced anxiety- and depressive-like behaviors (Kochalska et al., 2020; Jarosz et al., 2024). Importantly, these effects were abolished following vagotomy, indicating a crucial role of vagus nerve-mediated communication between the gut and brain in the context of GABA signaling (Liu and Forsythe, 2021). Additionally, microbially derived GABA has been shown to influence intestinal barrier integrity and modulate immune function (Conn et al., 2024). Although GABA itself does not cross the blood–brain barrier in significant amounts (Kakee et al., 2001), the interplay between gut microbial GABA production and the host’s neuroendocrine signaling—particularly the communication between the gut, the HPA axis, and the autonomic nervous system—remains a promising area of investigation, especially in relation to psychobiotics—live organisms that, when ingested in adequate amounts, confer mental health benefits. These findings underscore the therapeutic potential of targeting GABAergic activity through microbiota-based interventions in anxiety, depression, and stress-related disorders.

### 2.5 Role of gut bacteria-derived vitamins in mental health

Bacterially derived vitamins, particularly those from the B-complex group and vitamin K2, contribute to processes including neurotransmitter synthesis, energy metabolism, and neuroprotection. Emerging research suggests that deficiencies in these vitamins—often influenced by gut dysbiosis—may be linked to mood disorders, cognitive decline, and other psychiatric symptoms. While the underlying mechanisms vary, their importance in supporting brain health is increasingly recognized. A summary of these vitamins, their microbial sources, and mental health relevance—acknowledging that observed deficiencies often reflect overall systemic levels rather than microbiota-specific causes—is provided in **Table 1**.

**Table 1:** Microbially produced vitamins and their roles in cognitive function and mental health.

Vitamin	Producing bacteria	Role in mental health	References
Vitamin K2 (Menaquinone)	*Lactococcus*, *Bacillus*, *Bacteroides*, *Enterobacter*, *Escherichia coli*	Involved in lipid metabolism, neuroprotection, and anti-inflammatory processes. Deficiency may impair cognitive function.	(Walther and Chollet, 2017; Tarkesh et al., 2022)
Vitamin B1 (Thiamine)	*Lactobacillus fermentum*, *Enterococcus faecium*	Essential for nervous system function. Deficiency linked to dementia, depression, anxiety, and Wernicke-Korsakoff syndrome, especially in chronic alcohol use.	(Gibson et al., 2016; Linares et al., 2017)
Vitamin B2 (Riboflavin)	*Lactobacillus*, *Bifidobacterium*, *Escherichia coli*	Supports cerebral energy metabolism and reduces oxidative stress. Deficiency associated with mental fatigue and mood disturbances.	(Powers, 2003; Thakur et al., 2016; Averianova et al., 2020)
Vitamin B3 (Niacin)	*Bacteroides*, *Prevotella*, *Fusobacterium*	Critical for serotonin synthesis and brain energy metabolism. Deficiency can lead to irritability, depressive states, and cognitive decline.	(Prousky, 2010; Chand and Savitri, 2016)
Vitamin B5 (Pantothenic Acid)	*Bacteroides*, *Enterobacter*, *Saccharomyces cerevisiae*	Low levels correlate with fatigue and increased stress sensitivity. Functions as a precursor for coenzyme A, essential in energy metabolism and synthesis of neurotransmitters such as acetylcholine; low levels correlate with fatigue and increased stress sensitivity.	(Xu et al., 2020; Guo et al., 2023)
Vitamin B6 (Pyridoxine)	*Bacteroides fragilis*, *Lactobacillus*, *Bifidobacterium*	Vital for the synthesis of serotonin, dopamine, and GABA. Deficiency is closely linked to depression and anxiety symptoms.	(Calderón-Guzmán et al., 2004; Uebanso et al., 2020)
Vitamin B9 (Folate)	*Lactobacillus*, *Bifidobacterium*	Crucial for neurotransmitter production such as serotonin, dopamine, and norepinephrine, DNA synthesis, and mood regulation. Deficiency is strongly associated with major depressive disorders.	(Cicero and Minervino, 2022; Nara et al., 2023)
Vitamin B12 (Cobalamin)	*Propionibacterium freudenreichii*, *Lactobacillus reuteri* (limited synthesis)	Essential for neuronal integrity and mood. Deficiency may cause depression, memory impairment, and psychotic symptoms.	(Xu et al., 2018; Reis da Silva, 2024)

### 2.6 Role of microbial metabolites

In addition to directly influencing neurotransmitters, the gut microbiota produces a variety of metabolites that can impact brain function and mental health. The microbiome metabolome may act as a nexus of body-mind health. Among these metabolites, SCFAs and tryptophan metabolites play particularly significant roles:

- *SCFAs*, which include acetate, propionate, and butyrate, are key metabolites produced by the gut microbiota through the fermentation of dietary fibers (O'Riordan et al., 2022; Fock and Parnova, 2023). SCFAs can cross the blood–brain barrier (BBB) via monocarboxylate transporters located on endothelial cells. Once in the CNS, they can influence BBB integrity by increasing the expression of tight junction proteins, thereby contributing to the maintenance of the selective permeability of the BBB. This process is critical for preventing neuroinflammation because it limits the passage of peripheral immune mediators into the brain and protects neural tissue from harmful substances (Silva et al., 2020). In addition to their role in BBB maintenance, SCFAs modulate neuroinflammatory responses. They affect glial cell morphology and function, leading to alterations in the levels of neurotrophic factors, which are essential for neurogenesis and neuronal homeostasis. Moreover, SCFAs also contribute to the biosynthesis of neurotransmitters, including serotonin, by influencing the availability of their precursors and modulating the expression of related enzymes (O'Riordan et al., 2022). The impact of SCFAs on the gut–brain axis underscores the importance of considering dietary fiber intake in the context of mental health conditions. By promoting the production of SCFAs, a fiber-rich diet may support BBB integrity, help modulate neuroinflammation, and influence neurotransmitter synthesis, collectively contributing to improved cognitive function and mood regulation.
- *Tryptophan metabolism*: Tryptophan undergoes diverse microbial and host-driven transformations that extend far beyond serotonin synthesis. These pathways produce metabolites such as kynurenine and indole derivatives, which can influence immune regulation, oxidative stress, and gut–brain signaling, with emerging relevance to neurodevelopment and psychiatric disorders (Bosi et al., 2020). A substantial portion of dietary tryptophan is catabolized via the kynurenine pathway, producing metabolites such as kynurenine, quinolinic acid, and kynurenic acid—compounds that are closely linked to neuroinflammation and depressive disorders (Keszthelyi et al., 2009; Lukić et al., 2022). For example, elevated levels of quinolinic acid, a neurotoxic metabolite, have been observed in patients with major depressive disorder and are thought to contribute to glutamatergic dysregulation and neuronal damage (Busse et al., 2015; Öztürk et al., 2021). The gut microbiota can significantly influence this metabolic routing by shaping the host’s immune environment. Specifically, microbial components such as lipopolysaccharides (LPS) from gram-negative bacteria can activate immune responses and lead to the release of proinflammatory cytokines such as interferon-γ (IFN-γ) and tumor necrosis factor-α (TNF-α) (Michlewska et al., 2009). These cytokines, in turn, increase the activity of indoleamine 2,3-dioxygenase (IDO), a host enzyme that diverts tryptophan metabolism away from serotonin synthesis and toward the kynurenine pathway. This shift results in the production of neuroactive metabolites—such as quinolinic acid and kynurenic acid—which are associated with neuroinflammation, excitotoxicity, and mood dysregulation in individuals with disorders such as depression. Thus, while the microbiota itself may not directly modulate IDO, it influences the immune signals that govern its expression, illustrating the indirect but critical role of microbial–host immune interactions in brain function (Gao et al., 2018; Riazati et al., 2022). Additionally, certain commensal bacteria, such as *Lactobacillus reuteri* and *Clostridium* sp*orogenes*, metabolize tryptophan into indoles and their derivatives, such as indole-3-aldehyde, which activate the aryl hydrocarbon receptor (AhR), a pathway involved in immune regulation and neuroprotection (Salminen, 2023). Disruptions in the balance between serotonin synthesis and kynurenine pathway activity can shift the neurochemical environment toward a proinflammatory and neurotoxic state, which has been shown to contribute to cognitive dysfunction, mood disorders, and increased susceptibility to conditions such as depression and schizophrenia (Muneer, 2020; Correia and Vale, 2022). These findings underscore the important connection between microbial tryptophan metabolism and central nervous system function, underscoring its potential importance in mediating mental health.

## 3 Microbiota and psychological disorders

The influence of the gut microbiota extends beyond digestion and immune function, with a growing body of research showing a link between the microbiota and mental health and neurological function. Growing evidence (**Table 2**) suggests that dysregulation in the gut microbiome) suggests that gut dysbiosis contributes to the pathophysiology of various psychological disorders. Through mechanisms such as chronic low-grade inflammation (characterized by sustained elevation of proinflammatory cytokines), disruption of neurotransmitter pathways, and immune system dysregulation (including both hyperactive and insufficient immune responses), dysbiosis has been linked to conditions such as depression, anxiety, schizophrenia, ASD, and bipolar disorder. This section explores the relationship between the microbiota and these psychological disorders, shedding light on the potential mechanisms involved.

**Table 2:** Microbiota and psychological disorders.

Disorder	Microbiota alterations	Key mechanisms	Functional consequences/clinical relevance	Potential microbial influences	References
Depression & Anxiety	Reduced microbial diversity, decreased *Lactobacillus* and *Bifidobacterium*	Increased inflammation, serotonin dysregulation, gut permeability (“leaky gut”)	Linked to altered tryptophan–kynurenine metabolism and increased depressive symptoms; probiotic restoration improves mood	*Lactobacillus*, *Bifidobacterium* (support serotonin), proinflammatory bacteria (increase cytokines)	(Foster et al., 2017; Cryan et al., 2019)
Schizophrenia	Increased proinflammatory bacteria, reduced beneficial microbes	Neuroinflammation, altered dopamine signaling, oxidative stress	Dysbiosis correlates with cognitive impairment and dopamine-related signaling dysfunction	*Escherichia coli* (dopamine precursor), SCFA-producing bacteria (cognitive function)	(Rodrigues-Amorim et al., 2018; Zheng et al., 2019; Kelly et al., 2021)
Autism Spectrum Disorder (ASD)	Decreased diversity, increased *Clostridia* species	Gut permeability, altered neurotransmitters (GABA, serotonin), immune dysfunction	Altered microbial metabolites correlate with behavioral rigidity and social deficits	*Clostridia* (produce neurotoxic metabolites), *Bacteroides* (GABA production)	(Vuong and Hsiao, 2017; Sharon et al., 2019)
Bipolar Disorder	Altered microbiota composition, increased inflammatory markers	Chronic inflammation, gut-brain axis disruption, neurotransmitter imbalance	Dysbiosis associated with manic episodes and systemic inflammation	Probiotic strains (potential mood stabilization), inflammatory microbes (increase IL-6, CRP)	(Manchia et al., 2019; Nikolova et al., 2021)
Obsessive-Compulsive Disorder (OCD)	Lower microbial diversity, reduced *Faecalibacterium* and *Lactobacillus*	Disrupted serotonin pathways, gut-brain axis dysfunction, increased oxidative stress	Reduced anti-inflammatory and serotonin-modulating microbes correlated with OCD symptom severity	*Lactobacillus* (modulates serotonin), *Faecalibacterium* (anti-inflammatory effects)	(Januszewski et al., 2024)
Post-Traumatic Stress Disorder (PTSD)	Dysbiosis with reduced beneficial microbes	HPA axis dysregulation, increased gut permeability, inflammation	Dysbiosis linked to elevated cortisol, anxiety, and HPA dysregulation	SCFA-producing bacteria (gut barrier support), *Lactobacillus* (stress resilience)	(Leclercq et al., 2016; MacKay et al., 2024)

### 3.1 Depression and anxiety: dysbiosis, inflammation, and serotonin pathways

Depression and anxiety disorders are among the most prevalent mental health conditions, and a growing number of human studies have identified significant associations among the gut microbiota composition, microbe-targeted interventions, and these disorders. Case–control studies have shown that individuals with depression and anxiety often exhibit increased pro-inflammatory bacteria and reduced SCFA-producing taxa (Liang et al., 2018; Du et al., 2020; Simpson et al., 2021; Mitrea et al., 2022). Studies have demonstrated that individuals with depression often have reduced microbial diversity and depletion of beneficial genera such as *Lactobacillus* and *Bifidobacterium*. Notably, FMT experiments have provided compelling causal evidence: when the gut microbiota from depressed human donors or mice subjected to chronic stress are transferred into germ-free or antibiotic-treated rodents, the recipients develop anxiety- and depression-like the behaviors of the donors. These animals also exhibit increased neuroinflammatory markers—such as elevated hippocampal IFN-γ, TNF-α, and IDO1—as well as changes in microbial taxa (e.g., decreased *Lactobacillus* and increased *Bacteroides*) that parallel donor profiles (Romijn et al., 2017; Han and Kim, 2019; Jang et al., 2019; Li et al., 2019). This body of evidence strongly supports a link between dysbiosis and behavioral phenotypes and highlights the mechanistic relevance of gut–brain–immune interactions in depression and anxiety.

Inflammation has been strongly implicated in depression and anxiety, with increased levels of proinflammatory cytokines, such as interleukin-6 (IL-6) and TNF-α, observed in affected individuals (Elgellaie et al., 2023). Dysbiosis contributes to this inflammatory state by disrupting immune homeostasis primarily through increased intestinal permeability, which allows bacterial components such as LPS to translocate into the systemic circulation. This translocation triggers a systemic immune response characterized by elevated proinflammatory cytokines, which can, in turn, influence brain function and behavior through signaling along the gut–brain axis (Rutsch et al., 2020). Additionally, a reduction in serotonin-producing microbes can negatively impact mood regulation, potentially contributing to the onset of depressive and anxious symptoms (Sarkar et al., 2020; Li et al., 2025).

### 3.2 Schizophrenia: gut–brain connection in psychotic disorders

Schizophrenia is a severe psychiatric disorder characterized by hallucinations, delusions, and cognitive impairments (McCutcheon et al., 2023). While its exact cause remains unclear, research suggests that imbalances in the gut microbiota may contribute to disease progression (Ma and Song, 2023). Studies comparing the microbiomes of individuals with schizophrenia to those of healthy controls have revealed significant differences in microbial composition, with an increase in proinflammatory bacteria, including *Eggerthella*, *Lactobacillus*, and *Veillonella*; a decrease in potentially beneficial bacteria, such as *Faecalibacterium* and *Roseburia;* and a reduction in beneficial microbes, including *Coprococcus* and *Blautia* (Yan et al., 2022; Gokulakrishnan et al., 2023).

The role of the gut microbiota in schizophrenia may be linked to neuroinflammation, oxidative stress, and altered neurotransmitter activity (Carlessi et al., 2021). Gut microbial metabolites, such as SCFAs, have been implicated in schizophrenia through both developmental and symptomatic pathways. Early-life alterations in SCFA production—particularly butyrate production—have been associated with neurodevelopmental changes that may predispose individuals to schizophrenia. Additionally, during the onset of symptoms, reduced SCFA levels have been linked to increased gut permeability, systemic inflammation, and disrupted blood–brain barrier integrity, all of which may contribute to the cognitive and behavioral symptoms characteristic of the disorder (Peng et al., 2022; Kowalski et al., 2023). FMT studies in animal models have demonstrated that transferring gut bacteria from individuals with schizophrenia to germ-free mice induces schizophrenia-like behaviors, further supporting the role of the microbiota in this disorder (Vasiliu, 2023).

### 3.3 Autism spectrum disorder: early-life microbiota development and behavior

ASD is a neurodevelopmental condition characterized by social communication deficits and repetitive behaviors (Bhat, 2021; Hirota and King, 2023). Mounting evidence suggests that the gut microbiome may play a role in autism, particularly during early development, when the microbiota is still forming (Vuong and Hsiao, 2017; Taniya et al., 2022). Autistic children often exhibit distinct gut microbiota profiles, broadly including reduced microbial diversity and an increased abundance of certain bacterial species, such as *Clostridia*, which are known to produce neurotoxic metabolites (Fattorusso et al., 2019; Zou et al., 2020).

One potential mechanism linking gut microbes to ASD involves alterations in gut permeability and immune function (Azhari et al., 2019). Studies have reported increased intestinal permeability, or a “leaky gut,” in children with ASD, which may allow bacterial metabolites, bacterial components themselves and inflammatory molecules to enter the bloodstream and affect brain development (Eshraghi et al., 2020; Al-Ayadhi et al., 2021). Additionally, disruptions in the microbial production of neurotransmitters, including GABA and serotonin, may contribute to ASD-related behavioral symptoms (Garcia-Gutierrez et al., 2020; Bamicha et al., 2024). These imbalances may influence excitatory-inhibitory signaling in the brain, potentially contributing to core behavioral symptoms such as social deficits, anxiety, and repetitive behaviors. Emerging interventions, such as probiotic supplementation, dietary modifications and FMT, have shown promise in improving gastrointestinal and behavioral symptoms in autistic children, further highlighting the influence of the microbiota on neurodevelopment (Tan et al., 2021).

### 3.4 Bipolar disorder: immune–microbiome interactions

Bipolar disorder is a mood disorder characterized by alternating episodes of mania and depression (McIntyre et al., 2020). Recent studies suggest that immune system dysfunction and alterations in the gut microbiota may contribute to the pathophysiology of this disorder (Levy et al., 2017). Individuals with bipolar disorder often exhibit increased levels of systemic inflammation, with elevated concentrations of inflammatory markers such as C-reactive protein (CRP) and IL-6. Dysbiosis may contribute to this inflammatory state by disrupting gut barrier integrity and triggering immune activation (Doney et al., 2022; Zhao N. O. et al., 2022).

In individuals with bipolar disorder, alterations in the gut microbial composition have been linked to dysregulation of serotonin and dopamine pathways—neurotransmitter systems that are central to mood stabilization and are commonly disrupted during manic and depressive episodes (González-Arancibia et al., 2019). Additionally, preliminary research suggests that probiotic supplementation may help reduce inflammation and stabilize mood symptoms in individuals with bipolar disorder, opening new avenues for microbiome-targeted therapeutic strategies (Zeng et al., 2022). These effects are thought to arise through several interconnected mechanisms: by enhancing gut barrier integrity and thereby reducing leaky gut, by downregulating proinflammatory cytokine production and modulating immune responses, and by influencing the synthesis and availability of key neurotransmitters such as serotonin, dopamine, and GABA—each of which plays a crucial role in mood regulation. Together, these pathways highlight the therapeutic potential of targeting the gut microbiome in the management of bipolar disorder (Borkent et al., 2025; Zeng et al., 2022).

### 3.5 Gut microbiota and obsessive-compulsive disorder

Although research on the gut microbiota in patients with OCD is still in its early stages, emerging evidence suggests a meaningful connection between microbial composition and OCD symptomatology (Turna et al., 2016). Clinical studies have revealed a reduction in microbial diversity among individuals with OCD, particularly a decrease in beneficial butyrate-producing genera such as *Oscillospira*, *Anaerostipes*, and *Odoribacter*, which are known to play key roles in maintaining gut barrier integrity and anti-inflammatory signaling (Turna et al., 2020). Concurrently, an increased abundance of proinflammatory taxa such as *Rikenellaceae*—notably *Alistipes*—has been reported, indicating a shift toward a more inflammatory microbial profile (Parker et al., 2020; Tavella et al., 2021). These microbial alterations may contribute to increased intestinal permeability and systemic inflammation, both of which have been implicated in neuropsychiatric disorders. In support of a potential causal role, FMT from individuals with OCD into germ-free mice has been shown to induce compulsive-like behaviors and alter neurochemical pathways in recipient animals, including elevated succinic acid levels associated with neuroinflammatory responses (Zhang et al., 2024). In preclinical models, probiotic treatment with *Lactobacillus casei* Shirota has been found to attenuate compulsive grooming behavior in rodents, normalize BDNF expression, and modulate serotonin receptor levels, showing comparable effects to those of fluoxetine—a standard pharmacological treatment for OCD (Sanikhani et al., 2020). Collectively, these findings suggest that gut dysbiosis may contribute to the pathophysiology of OCD through immune modulation, microbial metabolite production, and effects on neurotransmitter systems. While human studies remain limited, this growing body of literature points to the gut microbiome as a promising target for future therapeutic interventions in patients with OCD.

### 3.6 Gut microbiota and post-traumatic stress disorder

Animal and preliminary human studies increasingly suggest that PTSD may involve disturbances in the gut microbial composition, intestinal barrier integrity, and downstream neuroimmune and neurochemical signaling. In a mouse model of repeated social defeat stress (RSDS), which induces PTSD-like behaviors, researchers reported significant gut dysbiosis, reduced gut microbial diversity, and disrupted barrier function characterized by altered tight junction proteins and increased permeability (He et al., 2024; Voigt et al., 2025). These mice also exhibited a proinflammatory gut milieu, supporting the hypothesis that repeated trauma induces a cascade from microbial imbalance to systemic immune activation (Yadav et al., 2023).

In humans, systematic reviews comparing PTSD patients to trauma-exposed controls have linked the reduced abundance of protective taxa—such as *Actinobacteria*, *Lentisphaerae*, and *Verrucomicrobia*—with PTSD symptom severity (Winder et al., 2025). Studies of specific populations, such as firefighters, revealed that individuals without PTSD symptoms presented greater gut microbial diversity and higher levels of SCFA-producing species, such as *Alistipes putredinis*, which has been associated with resilience to stress (Yoo et al., 2025). Therapeutically, pilot human trials using prebiotic supplementation in veterans with PTSD revealed modest increases in SCFA-producing bacteria and reductions in pro-inflammatory taxa, along with attenuated stress responses and trends toward decreased symptoms (Voigt et al., 2025). In rodent experiments, the administration of *Lactobacillus reuteri* DSM 17938 improved intestinal integrity, reduced systemic inflammation (e.g., lowered CRP), and reversed reductions in cortical BDNF linked to PTSD-like behaviors (Petakh et al., 2024). A separate mouse model using single prolonged stress (SPS) revealed that probiotic treatment mitigated anxiety- and depression-like behaviors, restored BDNF expression, and improved gut morphology (Khan et al., 2025).

Together, these findings suggest that PTSD may be associated with gut dysbiosis, increased intestinal permeability, and systemic inflammation—factors through which microbial metabolites and immune signaling influence neurobiology and behavior. While causal human data remain limited, these studies support the potential for microbiome-based adjunctive therapies in PTSD patients.

## 4 Mechanisms of influence

**Figure 3:**
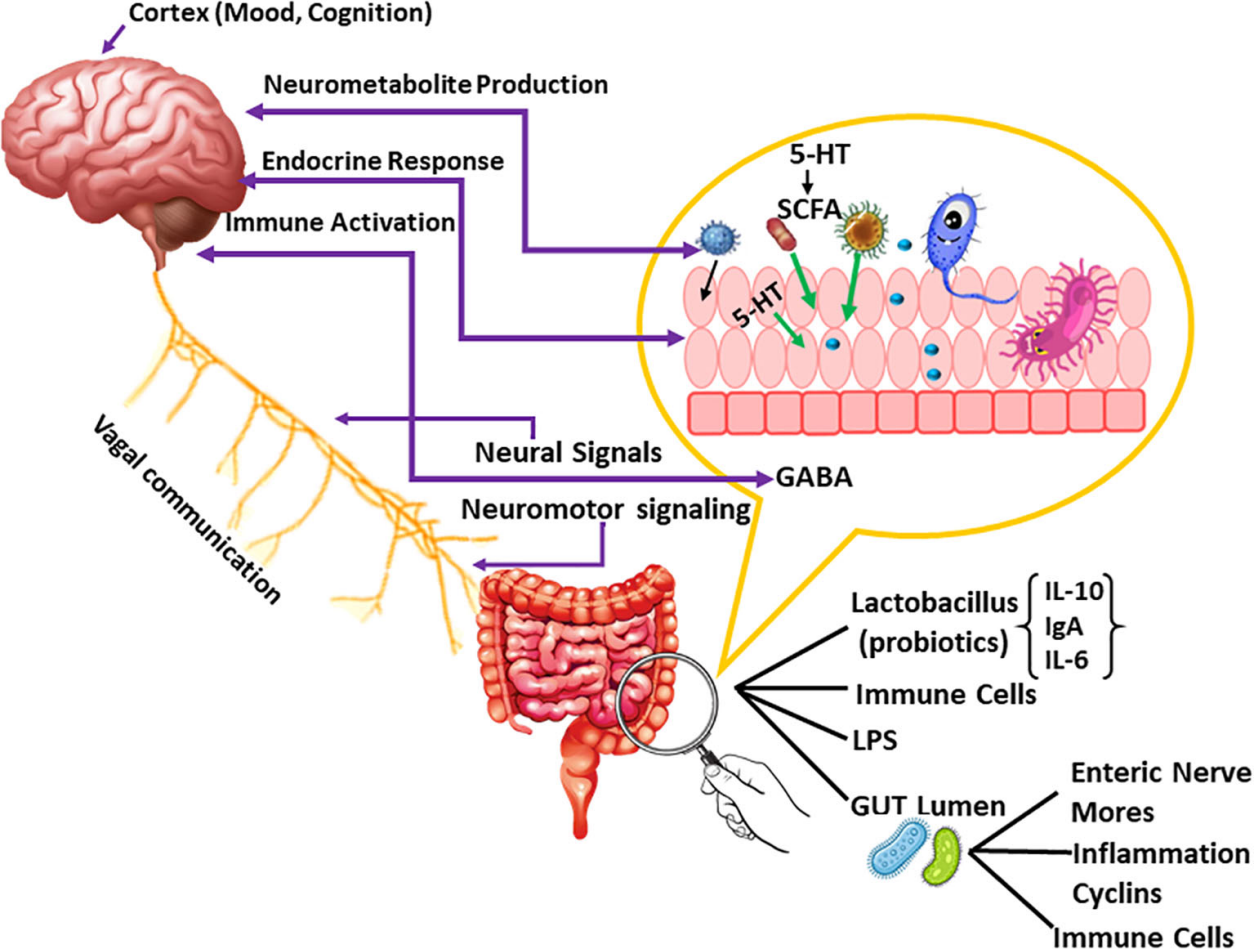
Mechanisms linking the gut microbiota to brain function and mental health. Microbial metabolites such as SCFAs and GABA, as well as serotonin and cytokines, affect neurometabolite production, immune activation, and endocrine responses through gut–brain communication.

The gut microbiota influences mental health through multiple interconnected pathways, including immune regulation and stress response modulation (Merlo et al., 2024). These mechanisms help explain how microbial composition affects brain function, behavior, and psychological well-being. Disruptions in these pathways due to dysbiosis can contribute to the development and progression of various mental health disorders. This section explores the key mechanisms by which the microbiota influences the brain and mental health, as illustrated in **Figure 3**.

### 4.1 Inflammation and immune modulation

The immune system plays a critical role in maintaining brain health, and the gut microbiota is an important regulator of immune function. A balanced microbiome supports immune homeostasis, whereas dysbiosis can trigger chronic low-grade inflammation, which has been implicated in several neuropsychiatric disorders, including depression, schizophrenia, and bipolar disorder (Mundula et al., 2022; Perdijk et al., 2024).

Microbial metabolites, such as SCFAs, help regulate inflammation by influencing immune cells (Parada Venegas et al., 2019). However, an imbalance in gut bacteria can lead to increased intestinal permeability, allowing inflammatory molecules such as LPS, which are components of the outer membrane of gram-negative bacteria and potent immune activators, to enter the bloodstream (Candelli et al., 2021). These proinflammatory signals can activate microglia, the brain’s immune cells, leading to neuroinflammation and contributing to cognitive and emotional disturbances (Qin et al., 2007; Skrzypczak-Wiercioch and Sałat, 2022). Peripheral immune mediators, such as cytokines and LPS, can cross the blood–brain barrier when its integrity is compromised or can signal indirectly via the vagus nerve and endothelial cells lining the cerebral vasculature (Huang et al., 2021).

Moreover, certain bacterial species modulate the production of anti-inflammatory cytokines (e.g., interleukin-10) and proinflammatory cytokines (e.g., interleukin-6, tumor necrosis factor-alpha), further influencing brain function (Strle et al., 2001). Chronic inflammation has been linked to alterations in neurotransmitter signaling, oxidative stress, and impaired neuroplasticity, all of which are associated with mental health disorders (Hassamal, 2023). Studies have shown that certain gut bacteria modulate the production of anti-inflammatory cytokines—such as interleukin-10 (IL-10)—as well as proinflammatory cytokines, including IL-6 TNF-α, which can significantly influence brain function (Steen et al., 2020). For example, *Faecalibacterium prausnitzii* is known to increase IL-10 secretion while simultaneously inhibiting NF-κB activation, thereby suppressing the production of inflammatory cytokines (IL-6 and TNF-α) (Alameddine et al., 2019). Conversely, dysbiosis characterized by an increased abundance of gram-negative taxa elevates LPS levels and stimulates immune responses that increase systemic IL-6 and TNF-α, both of which are markers linked to mood disorders such as depression and anxiety (Chen et al., 2024). Elevated IL-6 has also been shown to repress BDNF expression via epigenetic mechanisms in the brain, leading to impaired neuroplasticity and emotional regulation (Jehn et al., 2015). Collectively, these findings support the concept that microbial modulation of cytokine profiles—through both anti- and proinflammatory pathways—can induce neuroimmune changes directly associated with mental health outcomes.

### 4.2 Stress response and the HPA axis

The gut microbiota is a key modulator of the body’s response to stress, primarily through its interaction with the HPA axis (Rusch et al., 2023). The HPA axis regulates the body’s stress response system by controlling the release of cortisol, a hormone involved in mood regulation, energy metabolism, and immune function (Dedovic et al., 2009). In terms of mood regulation, persistent activation of the HPA axis—common in chronic stress—can lead to elevated cortisol exposure. Chronic cortisol elevation damages brain regions such as the hippocampus and prefrontal cortex, which are important for emotional regulation and cognitive control; this damage contributes to mood disturbances and depressive symptoms (Bertollo et al., 2025). With respect to immune function, cortisol exerts both acute anti-inflammatory effects and, under prolonged exposure, paradoxical immune dysregulation. While it suppresses proinflammatory cytokines (e.g., IL-6 and TNF-α) and dampens T-cell activity in the short term, chronic cortisol elevation may impair immune cell responsiveness and promote a shift toward Th2 dominance, reducing cellular immunity and increasing susceptibility to infections (Alotiby, 2024). Importantly, the gut microbiota and immune signals influence the HPA axis: microbial components such as LPS and elevated cytokines (e.g., IL-6) can activate the axis, increasing cortisol levels (Liu and Zhu, 2018; Bertollo et al., 2025).

Beneficial gut bacteria, such as *Lactobacillus* and *Bifidobacterium*, have been shown to modulate the stress response by reducing cortisol levels and promoting the production of GABA, an inhibitory neurotransmitter that helps counteract stress-induced excitability (Liu and Zhu, 2018; Tette et al., 2022; Zhao N. et al., 2022). Conversely, an imbalance in gut microbes can increase the release of stress hormones, increasing susceptibility to anxiety and depression-like behaviors (Nie et al., 2024).

Animal studies have demonstrated that germ-free mice exhibit exaggerated HPA axis responses to stress, further highlighting the role of the microbiota in stress regulation. Restoring beneficial microbes through probiotic supplementation has shown potential for modulating stress hormone levels and enhancing behavioral resilience, particularly in populations with HPA axis dysregulation. For example, in a murine model colonized with microbiota from patients with Cushing’s disease—a condition characterized by excessive cortisol production—probiotic intervention attenuated depression- and anxiety-like behaviors and normalized corticosterone levels. Similarly, human and preclinical studies have demonstrated that specific probiotic strains can influence the microbiota–gut–brain axis, thereby regulating cortisol output and improving stress-related outcomes (Wiley et al., 2017; Nie et al., 2024).

### 4.3 Effects of diet, probiotics, and prebiotics on mental health

Diet plays a crucial role in shaping the gut microbiome, which in turn influences mental health. Nutritional components such as fiber, polyphenols, and omega-3 fatty acids contribute to a balanced microbiota, supporting brain function and emotional well-being (Businaro, 2022). In contrast, diets high in saturated fats and refined sugars have been linked to dysbiosis, increased inflammation, and a greater risk of neuropsychiatric disorders (Leo and Campos, 2020).

Probiotics have been shown to positively impact brain function when this results in colonization in the gut. Specific probiotic strains, such as *Lactobacillus rhamnosus* and *Bifidobacterium longum*, help regulate neurotransmitter levels, particularly serotonin and GABA, and have been shown to be associated with reduced symptoms of depression and anxiety (Jarosz et al., 2024). These probiotics also play a role in reducing neuroinflammation, further supporting mental well-being (Meher et al., 2024).

Prebiotics, on the other hand, are nondigestible dietary fibers that serve as fuel for beneficial gut bacteria (Rezende et al., 2021). Compounds such as inulin and fructo-oligosaccharides promote the growth of bacteria that produce SCFAs (Birkeland et al., 2020), which have neuroprotective and anti-inflammatory properties. By fostering a healthier gut environment, prebiotics indirectly contribute to improved cognitive function and emotional stability. For example, Schmidt et al. reported that healthy volunteers who consumed galacto oligosaccharides (GOS) daily for three weeks presented a reduced cortisol awakening response and decreased attentional vigilance to negative stimuli, suggesting an anxiolytic effect (Schmidt et al., 2015). Similarly, a randomized controlled trial by Kato-Kataoka et al. demonstrated that prebiotic-containing fermented milk improved stress-related symptoms and sleep quality in medical students under academic stress (Kato-Kataoka et al., 2016).

Dietary patterns also play a significant role in mental health (Grajek et al., 2022). The Mediterranean diet—characterized by high intake of fiber, polyphenols, omega-3 fatty acids, and fermented foods—promotes microbial diversity and increases the abundance of beneficial taxa such as *Bifidobacterium* and *Lactobacillus*, as well as SCFA-producing species such as *Faecalibacterium prausnitzii* (Ghosh et al., 2020; Ventriglio et al., 2020). These microbial shifts are associated with reduced intestinal inflammation and improved blood–brain barrier integrity, both of which support cognitive function and emotional regulation (Jacka et al., 2017). In contrast, a Western diet—high in saturated fats, refined sugars, and low in fiber—has been shown to reduce microbial diversity and promote the expansion of proinflammatory bacteria such as *Bilophila wadsworthia* and *Alistipes (*David et al., 2014; Zhang et al., 2023). These findings highlight the importance of dietary interventions in promoting both gut and brain health, emphasizing the potential for nutritional strategies to help promote a healthy gut microbiome as a complementary treatment approach for managing psychological disorders.

## 5 Therapeutic potential

Growing evidence suggests that targeting the gut microbiome may provide novel therapeutic strategies for psychological disorders. Interventions such as probiotics, FMT, and diet-based approaches aim to restore microbial balance, reduce neuroinflammation, and regulate neurotransmitter production. These therapies have potential for improving symptoms of depression, anxiety, schizophrenia, and other mental health conditions.

### 5.1 Probiotics and psychobiotics

Probiotics have been studied for their role in mental health because of their ability to influence neurotransmitter production and regulate the immune response. Some probiotics, known as psychobiotics, have been shown to have specific effects on brain function by modulating serotonin, dopamine, and GABA levels (Sharma et al., 2021).

Psychobiotics also influence the HPA axis, which regulates the body’s response to stress. By promoting a balanced stress response and reducing cortisol levels, these beneficial microbes may help alleviate mood disorders (Bermúdez-Humarán et al., 2019). Clinical studies suggest that probiotic supplementation can improve emotional well-being, although its efficacy depends on factors such as strain specificity, dosage, and an individual’s baseline microbiome composition (Pirbaglou et al., 2016).

### 5.2 Diet-based interventions

The consumption of fiber-rich foods, fermented products, and essential nutrients such as omega-3 fatty acids can help maintain microbial balance and promote the production of beneficial metabolites (Nogal et al., 2021). Prebiotic-rich foods, including those containing inulin and fructo-oligosaccharides, provide nourishment for beneficial bacteria, stimulating their growth and increasing the production of SCFAs, which have neuroprotective and anti-inflammatory effects (Bornet and Brouns, 2002).

Additionally, certain dietary compounds, such as polyphenols found in plant-based foods, have been shown to support brain function by modulating gut bacteria and reducing oxidative stress (Shanmugam et al., 2022). Emerging research highlights the role of personalized nutrition in mental health, emphasizing the need for tailored dietary recommendations on the basis of an individual’s microbiome composition (Bianchetti et al., 2023).

## 6 Impact of antibiotic use and misuse on mental health

While antibiotics have been instrumental in combating infectious diseases, their overuse and misuse have raised significant concerns, particularly regarding gut microbiota disruptions and their subsequent effects on health. Antibiotics can dramatically alter the composition and diversity of the gut microbiota, leading to dysbiosis, which has cascading effects on neurodevelopment, behavior, and cognitive function (Dahiya and Nigam, 2023).

Dysbiosis induced by antibiotics leads to a reduction in beneficial bacteria and disrupts the production of neuroactive compounds such as SCFAs, GABA, serotonin, and dopamine, which are vital for supporting mental health (Guida et al., 2018; Bibi et al., 2025). Disruption of key microbial pathways—such as those involved in the synthesis of SCFAs, tryptophan metabolites, and neuroactive compounds such as GABA and serotonin—can impair neurotransmission and neuroplasticity. This microbial dysregulation has been associated with increased vulnerability to anxiety, depression, and cognitive decline through mechanisms involving heightened neuroinflammation, oxidative stress, and altered brain-derived neurotrophic factor (BDNF) signaling (Kelly et al., 2015; Dinan and Cryan, 2017; Mohajeri et al., 2018). Notably, early-life exposure to antibiotics is especially important. The first year of life represents a critical window for the establishment of a healthy gut microbiome and brain development (Fox et al., 2022). Several studies have shown that antibiotic exposure during this period is associated with an increased risk of behavioral problems and poorer cognitive outcomes later in childhood (Slykerman et al., 2023). Furthermore, large-scale population studies have identified links between early antibiotic use and increased risk of psychiatric disorders, including depression and anxiety, later in life (Lavebratt et al., 2019).

The mechanisms underlying these effects are multifaceted. Antibiotics can increase intestinal permeability (“leaky gut”), allowing microbial components or stimulating peripheral inflammation to enter systemic circulation and influence brain function (Chancharoenthana et al., 2023). In parallel, antibiotics may also modulate the HPA axis by disrupting the gut microbiota, which plays a key role in regulating stress responses. Such microbial disturbances can impair the production of microbial metabolites (e.g., SCFAs), reduce anti-inflammatory signaling, and increase gut permeability—ultimately leading to heightened activation of the HPA axis and potential neuroendocrine imbalances (Wu et al., 2020). Given these concerns, it is crucial to adopt judicious antibiotic use, especially during infancy and childhood. Clinical approaches should weigh the therapeutic benefits of antibiotics against potential long-term consequences for health. Furthermore, probiotic supplementation during or after antibiotic therapy is being explored as a strategy to restore microbiome balance and mitigate neuropsychiatric risk (Arslanova et al., 2021; Thabet et al., 2024).

## 7 Future directions and challenges

While research on the role of the gut microbiome in mental health has shown promise, significant challenges remain in translating these findings into effective clinical applications. Many studies rely on small sample sizes, animal models, or correlational designs, which limits the ability to draw causal conclusions about the efficacy of microbiome-targeted interventions. While concerns about long-term safety are particularly relevant for more invasive approaches, such as FMT, less invasive strategies, such as probiotics, are generally regarded as safe and may offer a favorable risk profile compared with conventional pharmacological treatments. Alterations in the composition of the gut microbiota have been consistently observed across various psychiatric disorders, including depression, anxiety, schizophrenia, ASD, and bipolar disorder. These changes are often characterized by reduced microbial diversity, increased abundance of proinflammatory taxa, and depletion of beneficial microbes.

While the observable associations between gut dysbiosis and mental health disorders are robust, the question of whether microbial alterations cause neurological symptoms or are secondary to them remains a critical knowledge gap in the field. Traditional cross-sectional and case-control studies dominate the literature, thereby limiting our capacity to infer true causality. Recent advances, however, offer promising routes toward disentangling directionality. For example, a Mendelian randomization (MR) study analyzing genome-wide data for gut microbiota taxa and psychiatric disorders identified bidirectional causal relationships: specific genera (e.g., *Bacteroides*, *Marvinbryantia*) influenced brain structure and psychiatric risk, while certain brain-region changes fed back to alter microbiota composition (Ye et al., 2025). Additionally, systematic reviews of FMT interventions have found that transferring microbiota from depressed or anxious humans into animals frequently induces behavioral changes consistent with mood disorders, suggesting that microbial composition can drive aspects of psychopathology (Chinna Meyyappan et al., 2020).Nonetheless, the repeated observation of distinct microbial signatures across different conditions supports the relevance of the gut–brain axis in psychiatric disease and underscores the need for longitudinal and mechanistic research to clarify causality. Advanced techniques such as metagenomics, metabolomics, and multi-omics approaches may help uncover the precise mechanisms underlying the gut–brain connection, allowing for more targeted therapeutic strategies. For example, a multi-omics study in first-episode schizophrenia patients revealed associations between microbial taxa, altered plasma metabolites (including GABA, tryptophan derivatives, and SCFAs), and disruptions in brain network connectivity, providing mechanistic insight into gut-to-brain signaling pathways (Wang Z. et al., 2024). Moreover, in patients with bipolar depression, concurrent fecal metagenomic profiling and serum metabolomics identified microbe-derived neuroactive compounds—such as kynurenic acid, B-vitamins, and GABA—that are correlated with changes in brain functional connectivity linked to mood regulation (Li et al., 2022). Additionally, the metabolomics of moderate–large cohorts with major depressive disorder has revealed dysregulated metabolites (e.g., bile acids and amino acid derivatives) that correlate with specific microbial genera, supporting integrated metabolic–microbiome relationships in depression pathophysiology (Hernández-Cacho et al., 2025; Liu et al., 2025). These sophisticated approaches not only help delineate causal and mechanistic pathways but also hold promise for informing more targeted microbiome-based therapies tailored to individual microbial–metabolite signatures.

Another major challenge is the considerable variability in the gut microbiota among individuals, which is influenced by genetics, diet, lifestyle, and environmental factors. This diversity limits the effectiveness of generalized interventions and highlights the need for personalized medicine approaches. Emerging technologies, such as microbiome sequencing and AI-driven analyses, could help identify microbial signatures associated with specific psychological disorders, leading to precision psychobiotics and customized dietary recommendations. However, the implementation of personalized microbiome-based therapies faces several hurdles, including high costs, accessibility issues, and the need for regulatory guidelines to ensure safety and efficacy. Despite these challenges, continued research and technological advancements hold the potential to revolutionize mental health care by integrating microbiome science into psychiatric treatment. As the field progresses, overcoming these obstacles will be crucial in unlocking the full therapeutic potential of microbiome-based interventions and developing more effective, individualized strategies for managing psychological disorders.

## 8 Linking global health and microbiome-driven mental health

Emerging evidence highlights that gut microbiota health is influenced not only by individual lifestyle choices but also by broader social, economic, and environmental factors. From a One Health perspective—which emphasizes the interdependence of human, animal, and environmental health—disruptions in local or global systems can significantly shape the gut microbiome composition and, consequently, mental health outcomes (Prescott et al., 2016; Maier and Al'Absi, 2017). In low-resource settings or areas affected by conflict, food insecurity and limited access to clean water are prevalent. These challenges, recognized within the United Nations Sustainable Development Goals (SDGs), are associated with malnutrition, exposure to environmental toxins, and increased antibiotic misuse, all of which contribute to gut dysbiosis.

Social inequality and political instability can restrict access to nutrient-rich diets and healthcare, further disrupting microbial balance and increasing vulnerability to stress-related mental health disorders. Additionally, environmental degradation, including loss of agricultural biodiversity and urban pollution, can alter microbial exposure during critical developmental windows, shaping the immune system and neurodevelopment in ways that may predispose individuals to psychological conditions (Singh et al., 2022). Addressing these systemic issues—through policies that promote food security, water sanitation, healthcare equity, and environmental protection—can support microbiome integrity and resilience, forming an upstream approach to mental health promotion on a global scale.

## 9 Summary

The complex relationship between the gut microbiome and the nervous system has emerged as a potential critical factor in fully understanding mental health and psychological disorders. The microbiome influences brain function through multiple pathways, including neural, immune, and metabolic mechanisms, with communication largely mediated by the vagus nerve, neurotransmitter modulation, and microbial metabolites such as SCFAs. Disruptions in microbial balance, or dysbiosis, have been linked to a range of psychiatric disorders, including depression, anxiety, schizophrenia, autism spectrum disorder, and bipolar disorder. Increasing evidence suggests that gut microbes contribute to neuroinflammation, immune dysregulation, and alterations in neurotransmitter production, all of which play key roles in the development and progression of mental health conditions.

With this growing body of evidence, microbiome-targeted therapies could offer promising new avenues for treatment. Probiotics, particularly psychobiotics, have demonstrated potential in regulating stress responses and improving mood by influencing the HPA axis and neurotransmitter pathways. FMT, although still used in psychiatry, has shown potential for restoring microbial diversity and decreasing symptoms in patients with neuropsychiatric conditions. Additionally, dietary interventions, including prebiotics and nutrient-rich foods, can support a healthy gut microbiome and contribute to mental well-being. However, despite these advancements, several challenges remain, particularly the need for large-scale clinical studies to confirm efficacy, the establishment of standardized treatment protocols, and a better understanding of the causal relationships between the microbiome and mental health. Personalized medicine approaches, leveraging microbiome sequencing and AI-driven analysis, may help tailor interventions to the individual’s microbiota, enhancing treatment effectiveness.

As research progresses, integrating microbiome science into psychiatric care could lead to a paradigm shift in mental health treatment. By addressing gut health as a key component of neurological and psychological well-being, future therapies may offer more effective, individualized, and holistic approaches to managing mental health disorders. However, overcoming current challenges—such as study limitations, variability in microbiota composition, and the need for regulatory frameworks—will be essential in translating these findings into practical, clinically viable solutions. The role of the microbiome in mental health remains an exciting and rapidly evolving field, with the potential to reshape our understanding of psychiatric disorders and open new doors for innovative treatments.
